# CoDeRS: a computing degree program recommender system using machine learning algorithms

**DOI:** 10.3389/frai.2026.1874202

**Published:** 2026-07-01

**Authors:** Christian Alexandra Rances, Rassel Aviel Sipe, Sherwin Baris, Adrian Lopez, Asia Dominic Rosaldo, Rex Bringula, Saida Ulfa

**Affiliations:** 1 College of Computer Studies and Systems, University of the East, Manila, Philippines; 2 Department of Computer Science, Linköping University, Linköping, Sweden; 3 Educational Technology Department, Faculty of Education, State University of Malang, Malang, Indonesia

**Keywords:** algorithm, artificial intelligence, degree program, IT education, recommendation system

## Abstract

This study reported the development and evaluation of CoDeRS (subsequently referred to as the software), a web-based recommender system designed to assist students in identifying suitable computing degree programs (e.g., Computer Science, Data Science, Animation, Game Development, and Information Technology). The software was developed using a six-stage process. The software generates tailored recommendations by integrating user profiles (e.g., hobbies, interests, and career aspirations), academic data, and personality types. Multinomial Naïve Bayes was applied to the user profiles, while content-based filtering was used to analyze academic data. The Big Five Inventory was used to determine the personality types of the students, and personality matching was done through cosine similarity. The recommendation engine of the software was built based on the data collected from the 352 computing students. Twenty senior high school students from diverse academic strands served as participants for the initial testing of the software and evaluated its quality in terms of usability, reliability, user experience, and overall satisfaction. Participants perceived that it is easy to navigate within the software (85.7%) and easy to use (81%), indicating its good usability. A large portion (76.2%) accepted the recommendations provided, and more than half expressed continuation of using it in the future. Performance metrics showed that accuracy was 81.6%, recall was 84.2%, and precision was 77.9%. The illustrative example, evaluation survey results, and performance metrics revealed that the software was effective. Thus, the software has the potential to be an effective technology to aid students in making informed computing degree program choices. Limitations and future works are also discussed.

## 1 Introduction

A mismatch between the students’ attributes (e.g., interests, hobbies, career aspirations, and academic strengths) and degree program choice is a perennial problem in the Philippines (Bringula et al., 2016). This mismatch is primarily attributed to uninformed decisions, which stemmed from unavailable information (Contini et al., 2018). It leads to high social costs (Parreño, 2023), such as wasted time and resources, lower academic performance, increased dropout rates, increased attrition, skill mismatch, and decreased trust in higher education institutions (Bringula et al., 2016; Contini et al., 2018; Casanova et al., 2018; O'Keefe et al., 2011). Even for students who completed a degree program that did not align with their genuine interests and aptitudes, there is a looming possibility of skills and interest mismatch, limiting their career or professional opportunities (Person et al., 2006).

One of the reasons for this mismatch is the students’ difficulty in aligning their hobbies, interests, career aspirations, and academic strengths with a degree program that best suits these characteristics. This led to students’ limited perspectives on which degree programs are suitable for their abilities and interests. For example, Bringula et al. (2016) found that computing students shifted due to a lack of interest in programming courses. Due to these pressing concerns, a computing degree program recommender system, called CoDeRS, was developed.

The software was developed as a data-driven solution to address the ongoing mismatch by aligning students’ profiles with appropriate computing degree programs. It can provide individualized and fact-based recommendations by combining academic data, personality traits, interests, hobbies, and career aspirations. This method gives students a better idea of which computing degree programs suit their profiles and strengths, thereby addressing the underlying causes of mismatch—uninformed decisions and the lack of information. By empowering students to make better decisions, CoDeRS reduces the likelihood that they will be dissatisfied with the program, transfer, or drop out altogether.

The development of the software is consistent with the increasing importance of artificial intelligence (AI)-driven transformation and digital innovation in educational systems. Recent studies have emphasized the necessity for educational institutions to leverage AI and data-driven technologies to support students’ degree program selection and overall academic success (Damanik et al., 2021; OECD, 2023; Borgonovi et al., 2025). In line with this context, AI-enabled educational software has emerged as a promising tool for assisting students in navigating complex academic degree program choices. Through the analysis of multiple attributes of students’ characteristics and the translation of these data into interpretable and verifiable degree program recommendations, the functionalities of the software are consistent with the goals of AI-enabled educational decision-support systems. More broadly, the software offers intelligent support services that could potentially enhance educational planning and promote informed decision-making. Such alignment is important because degree program choice ultimately influences workforce preparation and employability. In this regard, the software supports the broader objective of digital transformation in higher education, which emphasizes the use of data and digital technologies to improve decision-making processes and align educational pathways with evolving labor market demands (Mattiello and Wittberg, 2024).

This paper presents the development and evaluation of CoDeRS based on students’ profiles, personality types, and academic data. With this software, students could be given sufficient information to base their decisions on which computing degree program to pursue. The software would be able to help students, parents, and high school administrators actively participate in shaping the future of the youth. Specifically, the study aimed to meet the following objectives: (1) design and develop a software application that recommends a computing degree program based on various student data points, including academic data, interests, hobbies, career aspirations, and personality types, and (2) evaluate the quality of the system using the systems quality dimensions in terms of user experience, usability, reliability, user’s satisfaction, and performance metrics (e.g., accuracy, precision, and recall).

## 2 Theoretical basis

### 2.1 Degree program choice

Academic performance influences the degree programs that individuals choose to pursue. According to Rico-Briones and Bueno (2019), high academic achievement often leads students to select degree programs that align with their interests and academic strengths. Similarly, Berlingieri et al. (2023) highlighted the relationship between a student’s academic performance in specific subjects and their inclination toward related college programs. This finding is particularly evident among students who excel in Science, Technology, Engineering, and Mathematics (STEM) fields (Rafanan and De Guzman, 2020).

Furthermore, Gill et al. (2018) found that in the United States, students with strong academic backgrounds—especially in mathematics and science—are more likely to choose STEM majors. In Malaysia, Julaihi and Mohamadin (2024) discovered that personal decisions, family influence, employment opportunities, career aspiration, and academic qualifications were the most significant factors influencing degree choice. In the Philippines, Bringula et al. (2016) found that students enrolled in computing degree programs—such as Information Technology, Computer Science, Data Science, Animation, and Game Development—often shifted to other degree programs due to a lack of interest in programming. Conversely, those who remained in these programs typically did so because they had a strong interest in programming.

In a qualitative study of Cruz (2021), the author found that academically successful students typically choose degree programs that reflect their perceived competencies and career aspirations. In addition, Abay-Abay et al. (2024) observed that parental guidance, combined with academic performance, often steers Filipino students toward degree programs perceived as financially rewarding or prestigious. Overall, academic achievement, combined with cultural expectations, personal interests, and long-term career goals, plays a significant role in shaping students’ degree program choices. The summary of these findings is shown in Table 1.

**Table 1:** Summary of related works on degree program choice.

Cited studies	Key findings
Bringula et al. (2016)	Students in computing programs often shift to other degrees due to a lack of interest in programming, while those who stayed usually had a strong programming interest.
Rico-Briones and Bueno (2019)	High academic achievement often leads students to select degree programs that align with their interests and academic strengths.
Berlingieri et al. (2023)	Students’ academic performance in specific subjects influenced their inclination toward related college programs.
Rafanan and De Guzman (2020)	Students who excelled in STEM subjects were more likely to pursue STEM-related degree programs.
Gill et al. (2018)	In the United States, students with strong academic backgrounds—especially in mathematics and science—were more likely to choose STEM majors.
Julaihi and Mohamadin (2024)	In Malaysia, personal decisions, family influence, employment opportunities, career aspirations, and academic qualifications significantly influenced students’ choice of degree programs.
Cruz (2021)	Academically successful students were more likely to choose degree programs that match their perceived competencies and career aspirations.
Abay-Abay et al. (2024)	Parental guidance and academic performance influenced Filipino students to pursue degree programs considered financially rewarding or prestigious.

### 2.2 Students’ interests, hobbies, career aspirations, and personality types

Deciding on a career path heavily impacts an individual’s life, such as work, education, family, and leisure (Owusu et al., 2021). Selecting the appropriate college program is essential and should be accompanied by comprehensive planning and research. High school students begin to visualize their goals based on their identity, values, and the impact of peers, teachers, and parents (Lahoud et al., 2023). These social factors contribute to the motivation that encourages students to chase careers that align with their interests and abilities (Lahoud et al., 2023). When students realize their first program does not match their true interests, changing majors may take more time but often leads to a better fit with their goals and improved academic performance (Kamal et al., 2024).

Personality type also has a significant role in career choices; traits such as extraversion were linked to greater social involvement and engagement in college life (Brown and Hirschi, 2013). Research showed that group performance in organizations was affected by team personality composition and task nature (Kramer et al., 2014). In the educational context, students from different academic majors exhibited distinct personality types based on the Big Five model (Vedel, 2016). Students choose their academic programs based on their career ambitions, subject interests, or the pursuit of more accessible education (Skatova and Ferguson, 2014). Moreover, financial assistance from parents, student loans, or scholarships influences students’ degree choice (Bringula et al., 2016). A summary of the key findings discussed in this section is presented in Table 2.

**Table 2:** Summary of related works on students’ interests, hobbies, career aspirations, and personality types.

Cited studies	Key findings
Bringula et al. (2016)	Financial support sources—such as parental assistance, student loans, or scholarships—could influence students’ choice of degree programs.
Owusu et al. (2021)	Career path decisions significantly impact different aspects of an individual’s life, including work, education, family, and leisure.
Lahoud et al. (2023)	High school students begin forming career goals based on their identity, values, and the influence of peers, teachers, and parents; these social factors motivate students to pursue careers aligned with their interests and abilities.
Kamal et al. (2024)	Students who realize their initial degree program does not align with their interests may change majors; although this may take more time, it often leads to a better fit and improved academic performance.
Brown and Hirschi (2013)	Personality traits, such as extraversion, were associated with greater social involvement and engagement in college life, influencing career and academic choices.
Kramer et al. (2014)	Group performance in organizations was influenced by team personality composition and the nature of the tasks performed.
Vedel (2016)	Students in different academic majors were more likely to exhibit distinct personality types based on the Big Five personality model.
Skatova and Ferguson (2014)	Students select academic programs based on career ambitions, subject interests, or the perceived accessibility of the education.

### 2.3 Recommender systems and their usability

Before the development of recommender systems, content-heavy media such as books or movies became dependent on catalogs and basic search filters to assist users in finding what they needed. Recommender systems became known and acted as a software solution that utilizes data mining, filtering, and prediction algorithms (Kaklauskas et al., 2013). It offers personalized suggestions based on users’ preferences (Kaklauskas et al., 2013). The process of the recommender system involves the collection of user data, the prediction of preferences, and the delivery of relevant content. Currently, these technologies are intricately woven into numerous aspects of daily life, from recommending products and social connections to suggesting music (Fouad et al., 2025).

Recommender systems have also been found beneficial in the field of education (Lahoud et al., 2023; Kamal et al., 2024). They provide personalized assistance, guide students in navigating course options and degree choices, and help improve the academic decision-making process (Lahoud et al., 2023; Kamal et al., 2024). These systems have proven to transform education by offering personalized and intelligent guidance. By analyzing educational data, they recommend suitable academic paths to boost students’ motivation and performance (Lahoud et al., 2023; Kamal et al., 2024). Prior studies (Lahoud et al., 2023; Kamal et al., 2024) have extensively used factors such as demographics (e.g., language proficiency, income, and ethnicity), academic achievements, and career information (e.g., current occupation, career interests) for degree and course recommendation systems.

In another study, Shelake et al. (2025) developed DishaDoot, a career navigation platform designed to help students by recommending career paths based on academic performance, aptitude, interests, hobbies, and personality traits. It also includes scholarship recommendations and a support chatbot. The software was developed using the neural collaborative filtering (NCF) algorithm.

However, to the best of the authors’ knowledge, hobbies and personality types have not yet been included as input in recommender systems for degree choices. Furthermore, inputs for interests and career aspirations have been limited to predefined choices (Lahoud et al., 2023). This is an important issue to address because excluding hobbies and personality types, as well as limiting interests and career goals to predefined options, reduces the ability of the system to generate accurate, personalized, and meaningful recommendations for students. This limitation could be addressed by allowing users to provide open-ended responses, which can then be analyzed using natural language processing techniques. Table 3 provides a summary of the key findings in this section.

**Table 3:** Summary of related works on recommender systems and their usability.

Cited studies	Key findings
Lahoud et al. (2023)	Recommender systems in education provide personalized assistance and guide students in selecting courses and degree programs to improve academic decision-making. Existing systems often limit inputs for interests and career aspirations to predefined choices, which may reduce the personalization and accuracy of recommendations.
Kamal et al. (2024)	Educational recommender systems analyzed student data to recommend suitable academic paths, helping increase student motivation and performance.
Lahoud et al. (2023) and Kamal et al. (2024)	Previous degree and course recommendation systems commonly used factors such as demographics (e.g., language proficiency, income, and ethnicity), academic achievement, and career information (e.g., occupation, career interests).
Kaklauskas et al. (2013)	Recommender systems emerged as software solutions that use data mining, filtering, and prediction algorithms to provide personalized suggestions based on users’ preferences.
Fouad et al. (2025)	Recommender systems are widely integrated into daily life, recommending products, social connections, and media such as music.
Shelake et al. (2025)	Developed a career path recommendation system for students.

### 2.4 AI-driven transformation for education

The educational system could benefit from digital transformation, particularly through AI-enabled educational decision-support systems. These types of systems could improve equity, efficiency, and decision quality across all levels of education. From an ethical perspective, Floridi and Cowls (Benavides et al., 2020) argued that such AI systems must adhere to the principles of beneficence, non-maleficence, autonomy, justice, and explicability. These principles guide software developers in creating AI-enabled educational tools that are human-centered, transparent, and beneficial. Moreover, they require developers to ensure that algorithmic recommendations not only optimize outcomes but also remain aligned with fairness, human oversight, and the protection of student autonomy.

Complementing this ethical perspective, the OECD (Damanik et al., 2021) viewed education as a comprehensive digital ecosystem that requires the seamless integration of data, governance, infrastructure, and AI tools. Within this ecosystem, decision-support systems become valuable mechanisms for connecting student data with the goal of improving instructional, administrative, and career guidance processes. (OECD, 2023) further emphasized the need for AI adoption in education, arguing that AI should address persistent challenges such as dropout risks, learning disparities, and inequitable access to educational opportunities. To achieve this goal, a functional management system must be developed to integrate student data, career guidance services, and school administrators (Damanik et al., 2021). However, despite the potential benefits of these initiatives, several barriers remain. These include the lack of holistic implementation (Mattiello and Wittberg, 2024), governance and capacity gaps (Damanik et al., 2021; OECD, 2023), and cultural mismatches (Borgonovi et al., 2025) that may hinder technology adoption across different educational contexts.

## 3 Software description

### 3.1 Software development

CoDeRS is a web-based recommender system. It was developed through a six-stage prototype process model designed to make it both functional and easy to use (Maryani et al., 2022). This software development process model was deemed applicable to the development of CoDeRS because there is a need to fine-tune the model and the features of the recommender system. The process started with Requirement Gathering, where the team formed the design goals of the system and collected relevant student data. The next step involved the Quick Design phase, which included creating a paper prototype to gather early feedback and confirm the concept before moving on to actual implementation.

During the Building Prototype stage, a detailed, working version of the system was created, complete with a usable interface and content. The system was developed using a combination of programming languages. Python was utilized for the implementation of machine learning (ML) algorithms, data processing, and model training. JavaScript was used to manage backend logic and system functionalities. HTML and CSS were employed to design the front end (i.e., website) of the system.

Furthermore, the recommendation model was developed at this stage. To build the neighbors (i.e., the dataset containing the profiles of computing students), computing students from one university participated in the study. A sample size of 333 computing students was computed using Slovin’s formula (*N* = 2,000; *e* = 0.05). A total of 352 computing students (Information Technology = 236; Computer Science = 102; Data Science = 6; Entertainment Computing major in Animation = 4; and Entertainment Computing major in Game Development = 4) in one university in Manila participated in the study. The total number of students enrolled in Data Science is 11, in Animation is 10, and in Game Development is 12. These low enrollment figures account for the limited number of participants in these degree programs. The dataset served as the reference data against which the prospective student’s information was matched. The ethics review committee of the university approved the data collection.

The students’ profiles were collected in a spreadsheet. They were subjected to data preprocessing to extract the career aspirations, hobbies, and interests of the students. They were aggregated and saved as a text file. Stop words (e.g., “a,” “an,” “the,” etc.) were removed from the text file. Stemming was employed using Python. There were 26 unique words with a total word count of 663. These words comprised the corpus. The words “game” (*n* = 154), “play” (*n* = 101), “basketball” (*n* = 52), “watch” (*n* = 41), and “read” (*n* = 41) were the top 5 most appearing words in the corpus. Table 4 further shows the top 3 aspirations, hobbies, and interests of computing students per degree program. Among all the degree programs, Computer Science students had the highest number of career aspirations (*n* = 28) and hobbies (*n* = 76). Computer Science (*n* = 20) and Information Technology (*n* = 22) students had nearly the same number of interests.

**Table 4:** Top 3 aspirations, hobbies, and interests of computing students.

Profile	EMC-animation	EMC-game development	Computer science	Data science	Information technology
Aspirations	Creative arts Sports Film (*n* = 4)	Game developer Animation artist (*n* = 2)	Software Developer Game developer Programmer (*n* = 28)	Programmer Data scientist Entrepreneur (*n* = 6)	Web developer Software Developer IT Specialist (n = 26)
Hobbies	Playing games Drawing /Art Basketball (*n* = 6)	Playing games Watching movies Coding (*n* = 11)	Playing games Reading Listening to music (*n* = 76)	Reading Watching (anime, shows, etc.) Listening to music (*n* = 15)	Playing basketball Playing games Reading (*n* = 31)
Interests	Games Physical Education Science (*n* = 10)	Graphic Design Game Development Filmmaking (*n* = 9)	Coding Science Mathematics (*n* = 20)	Statistics Programming Socializing (*n* = 13)	Coding Science Research (*n* = 22)

**Figure 1:**
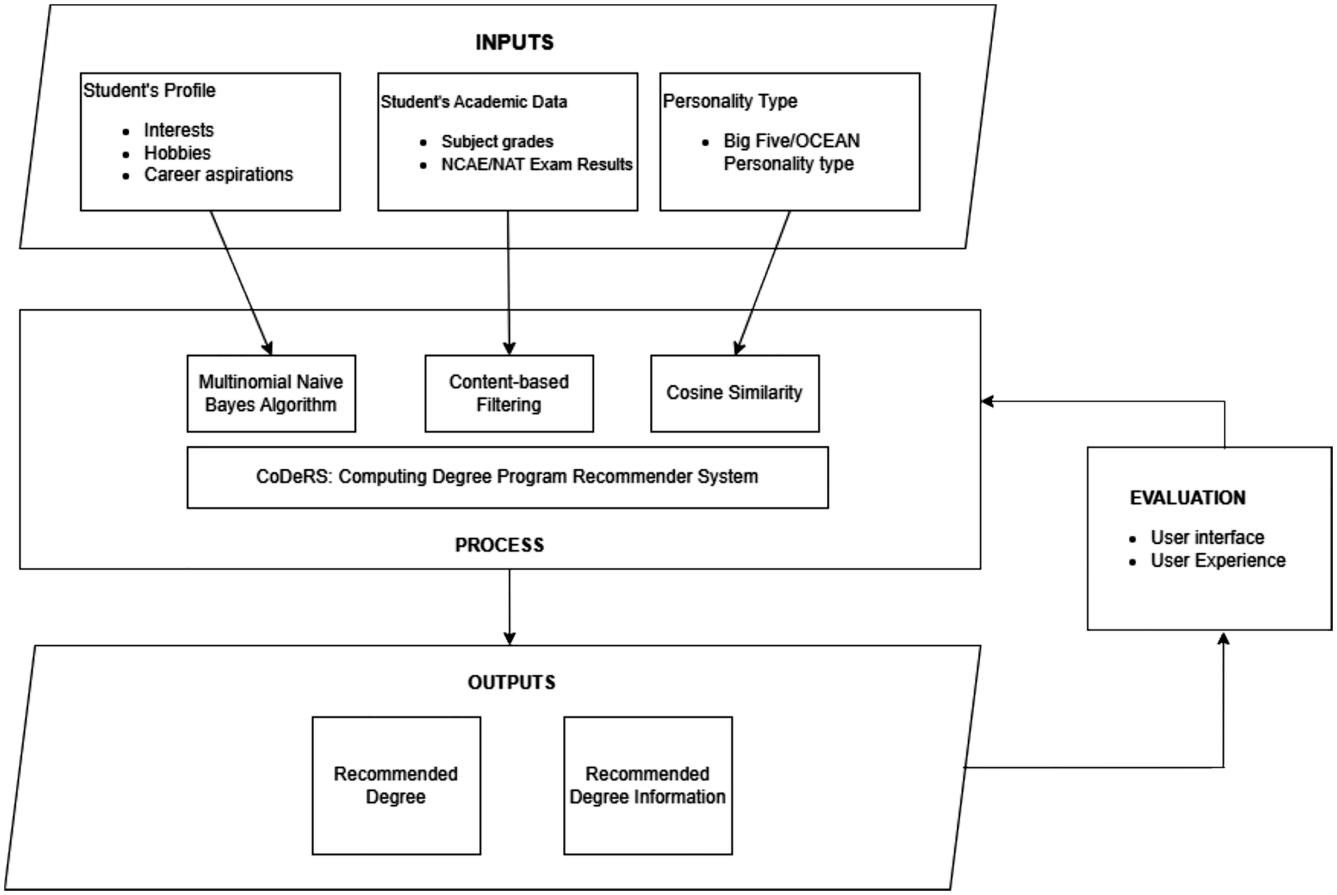
Conceptual framework guiding the development and evaluation of the software.

The inputs for the recommender system include students’ profiles, academic data, and personality types (Figure 1). The multinomial Naïve Bayes Algorithm was utilized for text classification of student profiles (Equation 1). Content-based filtering (CBF) was used to analyze the textual and academic data (grades and national assessment exam scores). The grades consisted of Math, Science, and English, as these are the three subjects used to determine admission into the program. Grades, NCAE (National Career Assessment Examination), and NAT (National Achievement Test) scores were expressed as percentages. The percentage ranges and verbal equivalents are as follows: 75: Did Not Meet Expectations, 75–79: Fairly Satisfactory, 80–84: Satisfactory, 85–89: Very Satisfactory, and 90–100: Outstanding (Department of Education, Policy guidelines on classroom assessment for the K to 12 basic education program, 2015). These national, standardized tests are aimed at assessing the academic performance of the students. The numerical academic performance indicators were transformed using rule-based discretization (Amatriain and Pujol, 2015). The percentage scores were mapped into ordinal categories based on predefined thresholds. This process converted continuous grade values into categorical representations. These categorical representations were then used as part of the feature set in the content-based filtering system. Finally, cosine similarity assessed the similarity of the student’s personality type [Equation 2; (Amatriain and Pujol, 2015)]. The personality type was measured using the Big Five Personality Inventory model. The software would recommend that users take a computing degree program if it is recommended by at least two of the algorithms.


$$P(w_{i}|c_{j})=\frac{\text{count}(w_{i},c_{j})+1}{(\sum_{w\in V}\text{count}(w,c_{j}))+|V|}$$ (1)

Where:

*P*(*w_i_|c_j_*): the probability of a word *i* falling into category *j*.

*count*(*w_i_,c_j_*): the number of query words that appear in a class or category.

Constant 1: a constant 1 is added to avoid zero values.

$\sum_{w\in V}\text{count}(w,c_{j})$ : The sum of all the words in the class or category c_j_.

Equation 1 The multinomial naïve Bayes formula (Damanik et al., 2021)


$$\text{Cosine Similarity}\;(x,y)=\frac{\left(x•y\right)}{\left\|x\|\|y\right\|}$$ (2)

Equation 2 Cosine Similarity, where x•y is the dot product of vectors x and y, and ||*x*|| and ||*y*|| are the magnitudes of vectors *x* and *y* (Amatriain and Pujol, 2015).

The Multinomial Naïve Bayes algorithm, cosine similarity, and CBF were selected for the recommender system for several reasons. In general, these methods are aligned with the objectives of the system and the types of data collected. The Multinomial Naïve Bayes algorithm was utilized to analyze textual data (i.e., career aspirations) because it performs well with categorical and text-based data (Alsanad, 2022). Cosine similarity was employed because it can represent personality traits as multi-dimensional vectors derived from a personality assessment instrument. It enables the system to determine how closely aligned two personality profiles are, regardless of differences in magnitude (Banik, 2018). Meanwhile, CBF was used because it can generate recommendations that align with prospective students’ academic strengths by comparing their profiles with those of current students stored in the database (Banik, 2018). Finally, these three algorithms were selected because they are relatively easy to verify, explain, and interpret, which supports transparency in the recommendation process.

Hybrid filtering and deep learning techniques were not yet implemented in this study due to limitations in the available dataset, the characteristics of the selected attributes, and the absence of user interaction data in the current system. The last component is typically required for collaborative filtering analysis. In summary, the selected algorithms were chosen because they are computationally efficient and perform well on relatively small to medium-sized datasets. These algorithms are suitable for the current stage of the development of the software. Compared with more complex algorithms (e.g., deep learning-based algorithms), the chosen methods require less training data, provide greater interpretability, and enhance transparency in the recommendation process (Larsson and Heintz, 2020).

Four experts evaluated the prototype before it was deployed. They were composed of three software developers and a software quality assurance specialist with at least 5 years of industry experience. They offered valuable suggestions for improvement. The system was refined multiple times during the Refining Prototype phase to enhance both the software’s accuracy and the user experience. Finally, the team launched it during the engineering product stage. At this stage, senior high school students utilized the software.

### 3.2 Software architecture

**Figure 2:**
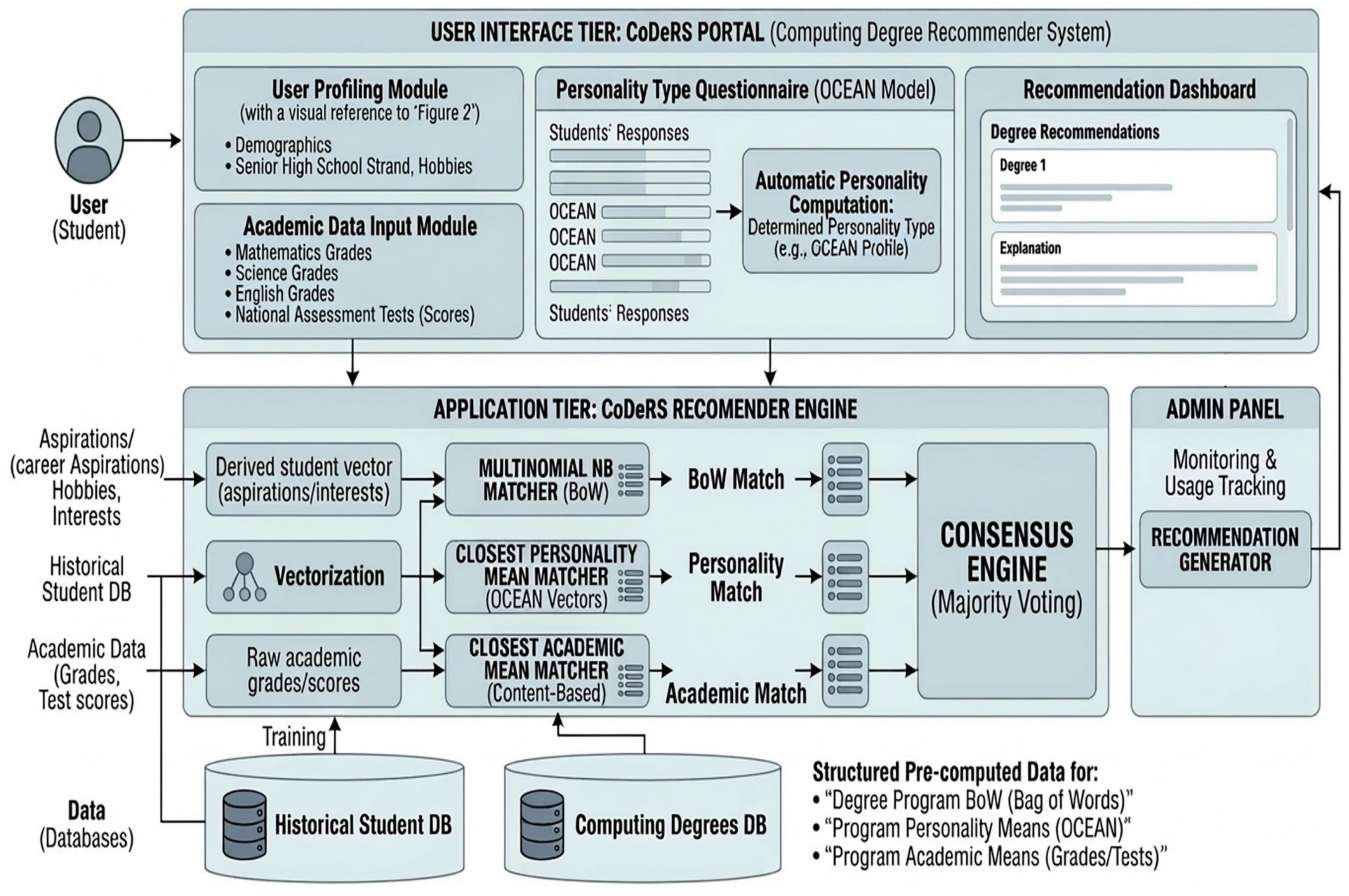
The system architecture of CoDeRS.

The CoDeRS architecture begins with a user interface tier (Figure 2). In this tier, it captured a prospective student’s profile, academic data, OCEAN personality traits, and career aspirations. These data were then processed in parallel by an application tier. The application tier employed three distinct algorithms discussed in the previous section. These algorithms independently matched the inputs against pre-computed computing degree requirements. Finally, a consensus engine employed majority voting on these individual matches to generate a single, tailored degree recommendation. An explanation of how the system arrived at this recommendation is shown on the student’s dashboard.

**Figure 3:**
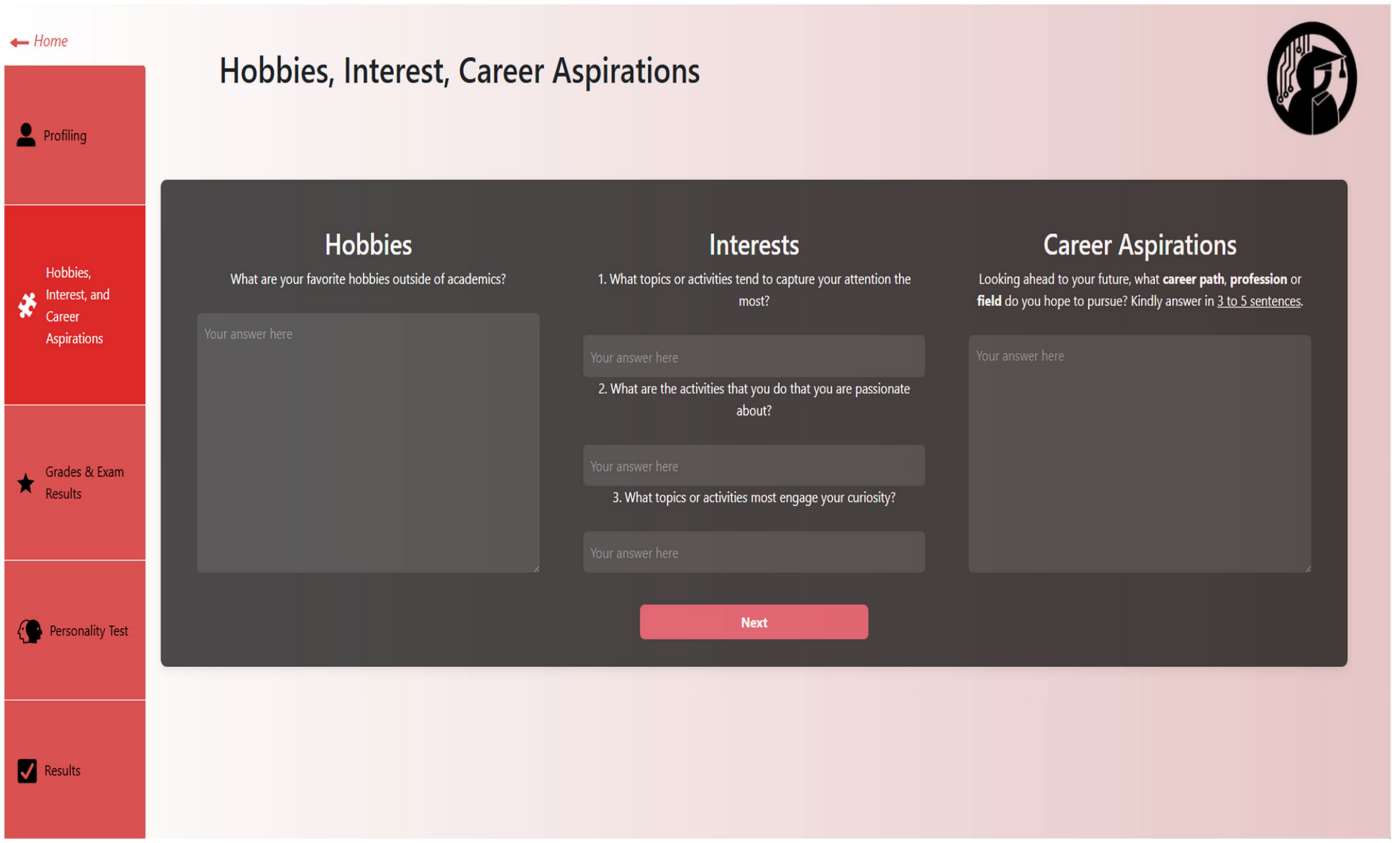
A screenshot of a module of the CoDeRS.

The software was comprised of different modules (or pages), including user profiling, academic data input, and personality types. The user profiling module allowed the users to input their demographics, senior high school strand, hobbies, interests, and career aspirations (Figure 3). The academic data input module would collect the prior subject grades in Mathematics, Science, and English, and the national assessment tests. The personality types questionnaire was also incorporated into the system and could automatically compute and subsequently determine the prominent personality type of the student.

### 3.3 Software functionalities

The software includes several key features and capabilities that work collaboratively to achieve the objectives of the study. Below are the features of the software.

- *Strand and demographics profile page*: This page collects basic user information such as age, gender, current senior high school strand (e.g., STEM, ABM, HUMSS), and monthly gross household income.
- *Hobbies, interests, career aspiration page*: This page allows students to input their hobbies (e.g., *What are your favorite hobbies outside of academics?”*), interests (e.g.,” *What topics or activities tend to capture your attention most?*,” *“What are the activities that you do that you are passionate about?,” and “What topics or activities most engage your curiosity?”*), and career aspirations (e.g., “*Looking ahead to your future, what career path, profession, or field do you hope to pursue?*”) (Figure 3). The data gathered supports the recommender system in suggesting a computing degree program that aligns with a student’s interests and career goals. These questions are also shown in Figure 3.
- *Grade and exam results page*: This page allows students to input their academic performances in core subjects like Math, Science, and English. These scores provide a measurable basis for evaluating students’ strengths and help the system recommend computing degree programs.
- *Recommendation engine*: Utilizing different algorithms, the software processes the user’s input and generates a computing degree program recommendation.
- *Results dashboard*: After analysis, the user is presented with a dashboard showing the recommended field, a brief explanation, and suggestions for further exploration in the selected track.
- *Admin panel*: Administrators can access a secure panel that enables them to monitor system usage.
- *Personality type test (OCEAN) page*: This page contains the 46 items of the OCEAN personality type (John and Srivastava, 1999). It could be answered on a scale of 1 (disagree strongly) to 5 (agree strongly). The five dimensions of personality in the Big Five personality model are Openness (e.g., “*Likes work that is the same every time.*”), Conscientiousness (e.g., “*Can be somewhat careless.”*), Extraversion (e.g., “*Tends to be quiet.*”), Agreeableness (e.g., “*Is helpful and unselfish with others*.”), and Neuroticism (*“Is relaxed, handles stress well.”*). The Cronbach’s alpha reliability coefficients were 0.81 for Openness, 0.82 for Conscientiousness, 0.88 for Extraversion, 0.79 for Agreeableness, and 0.84 for Neuroticism. The validity coefficients were 0.92 for Openness, 0.92 for Conscientiousness, 0.94 for Extraversion, 0.92 for Agreeableness, and 0.90 for Neuroticism (John and Srivastava, 1999).

### 3.4 Software testing

For initial testing, 20 senior high school (SHS) students from a university in Manila participated in the study. Purposive sampling was employed to select the participants. Four SHS teachers were requested to assist in the selection process. Only students who voluntarily agreed to participate were included in the study. The number of participants was limited due to the policy on non-disruption of classes and the constraints of a tight class schedule. Participation was restricted to students’ free time, ensuring that their classes were not affected. All six SHS strands were represented, including ABM (Accountancy, Business, and Management), HUMSS (Humanities and Social Sciences), and STEM. Most participants were Grade 11 students (76.2%), with 23.8% from Grade 12. Strand representation was diverse: ABM and STEM students each made up 28.6%, followed by GAS (23.8%), HUMSS (19%), and a smaller portion from TVL and Sports strands.

A researcher-made survey form was utilized to evaluate the quality of the software. The survey was based on the software quality dimensions in terms of user experience (e.g., “*The recommender system is easy to use*.”), usability (e.g., “*The software provides clear instructions.*”), reliability (e.g., “*I trust the program recommendations*.”), and user satisfaction (e.g., “*I am satisfied with my overall experience using the software*.”) (DeLone and McLean, 2003). All criteria have five items that could be answered using a scale of 1 (strongly disagree) to 5 (strongly agree). The survey form also included an open-ended question to gather respondents’ comments or suggestions for improving the software. Three researchers in the field of usability studies and software development validated the content of the instrument. Finally, accuracy, precision, and recall were computed to determine the performance of the recommender system [Equation 3; (Amatriain and Pujol, 2015)].


$$\begin{array}{l} \text{Accuracy}=(\mathrm{TP}+\mathrm{TN})/(\mathrm{TP}+\mathrm{TN}+\mathrm{FP}+\mathrm{FN})\\ \text{Precision}=\mathrm{TP}/(\mathrm{TP}+\mathrm{FP})\\ \text{Recall}=\mathrm{TP}/(\mathrm{TP}+\mathrm{FN}) \end{array}$$ (3)

Where TP, true positive; TN, true negative; FP, false positive; FN, false negative.

Equation 3 performance metrics (Amatriain and Pujol, 2015).

The software was implemented as a recommender system designed to generate a single degree program recommendation. Ranking-based metrics (e.g., Precision@K, Recall@K, and NDCG@K) were not deemed appropriate because these metrics typically require ranked recommendations and user interaction data. Accuracy, precision, and recall were used as evaluation metrics because the recommendation task was treated as a classification problem (i.e., determining which degree program is approximately most suitable for a student) (Amatriain and Pujol, 2015).

The existing dataset was split into training (70%) and test (30%) sets. It is worth noting that the dataset is considered suitable for approximating suitable degree programs for several reasons. The software was designed to identify similarity-based student–degree program matches rather than evaluate post-enrollment outcomes or optimal degree choices. Moreover, prior literature on degree choice, which currently provides the most appropriate basis for this purpose, offers a framework for approximating degree program selection (Rico-Briones and Bueno, 2019; Berlingieri et al., 2023; Rafanan and De Guzman, 2020; Cruz, 2021). Furthermore, a sufficiently large sample size may help reduce individual-level noise in the dataset and improve the accuracy of the observed patterns.

### 3.5 Illustrative example

**Figure 4:**
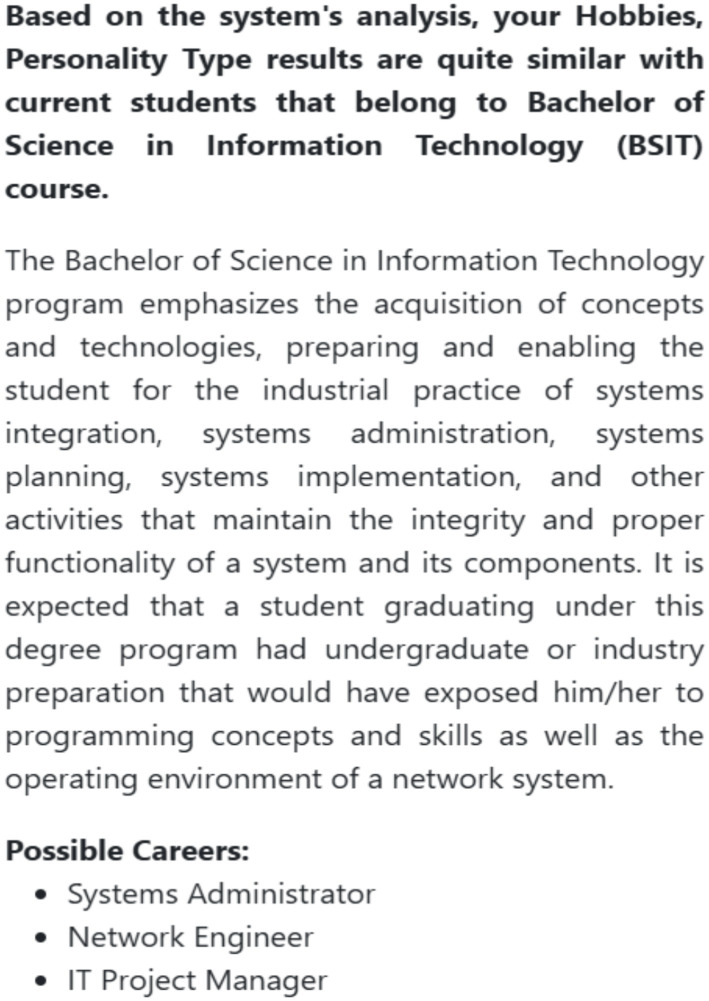
CoDeRS explaining its result.

The CoDeRS link can be accessed here: https://ml-recommender-system.onrender.com/. Figure 4 shows a sample output of the software. The software was able to determine whether the students’ attributes were suited to a computing degree program. For example, a student who belongs to the Technology-Vocational Livelihood-Information and Communication Technology strand (TVL-ICT) with different profiles was shown to be a good fit for the Information Technology degree program (Table 5).

**Table 5:** Sample student A persona.

Student A persona				
Strand			Age	Gender
TVL-ICT			18	Female
Monthly household gross income PHP 48,401—below PHP 84,600				
Interests			Hobbies	
Sports Badminton Basketball Animation			Reading manga Badminton	
Career aspirations “Data Science is probably the career path I wanted to pursue. It is new, and I was encouraged during the data science seminar as I love doing research activities, and I also look forward to learn a lot more from the course. I wanted to expand my knowledge about it ever since I was interested in the course.”				
Subject grades	Math 85–89	Science 90–100	English 90–100	NCAE/NAT Exam 90–100
Personality type			Agreeableness	
Recommended degree program			BSIT	

**Figure 5:**
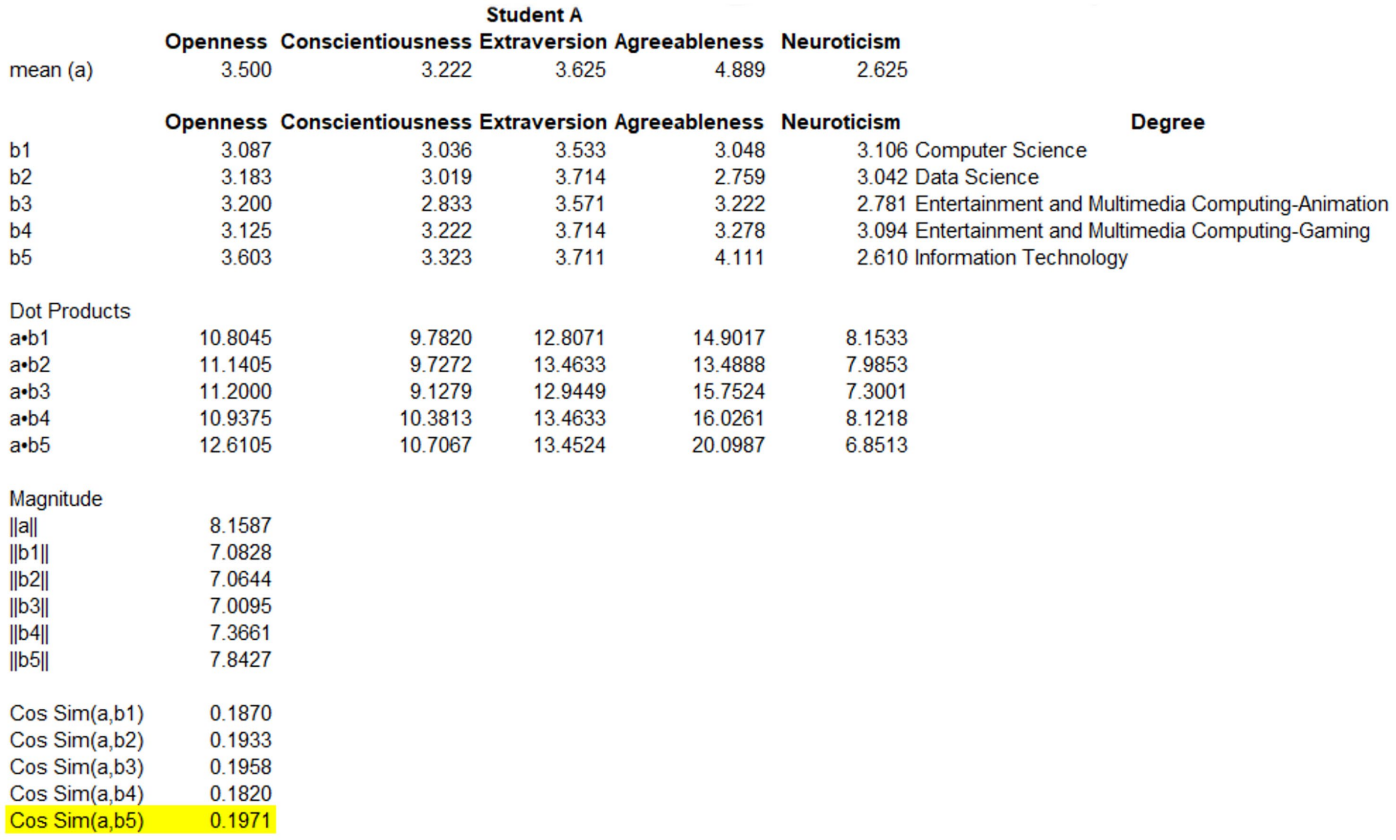
Implementation of cosine similarity for student A.

**Figure 6:**
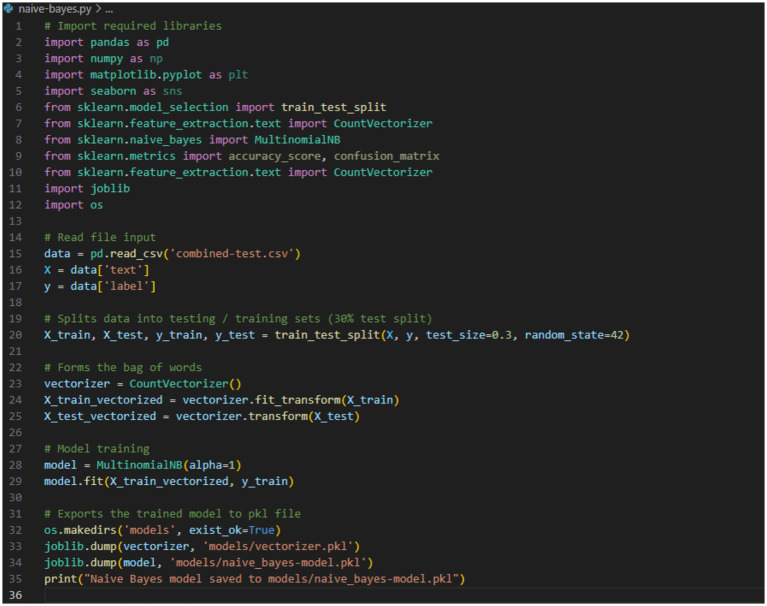
Implementation of multinomial naïve Bayes in python.

To provide readers with insight into how CoDeRS arrived at a suitable recommendation, a manual computation of cosine similarity and CBF was presented. Although several sample personas were validated, only one is presented in this paper for the sake of brevity. The cosine similarity showed that the personality type of Student A is closely related to that of students taking up the IT degree program (Figure 5). Meanwhile, performing a manual computation for skills using the Multinomial Naïve Bayes algorithm would be very tedious due to the diversity of words in the training data. Instead, a screenshot of the Python implementation of the Multinomial Naïve Bayes algorithm is shown in Figure 6. A built-in Python function was used to implement the Multinomial Naïve Bayes algorithm.

For the CBF component, the student’s academic data is mapped in Table 6. The values in Table 6 represent the students’ average grades and their corresponding equivalent ranges in the dataset. The results of the grade mapping for Student A are as follows: Math (85–89) → {GD, IT}, Science (90–100) → {CS}, English (90–100) → {CS, DS}, and NCAE/NAT (90–100) → {GD}. In this mapping, GD and CS appear twice. Therefore, based on Student A’s grades, GD and CS are approximately suitable degree programs. The first two algorithms, however, recommend IT. These recommendations override the CBF result. Thus, the final recommendation is IT. It was shown that the recommendation of CoDeRS aligns with manual computation and illustration. The software further explains why Student A is suited for the BS Information Technology degree program (Figure 4).

**Table 6:** Average grades and their equivalent ranges for students in different degree programs.

Degree	Math	Science	English	NCAE/NAT
Computer science (CS)	90.07 [90–100]	89.96 [90–100]	91.03 [90–100]	87.88 [85–89]
Data science (DS)	90.17 [90–100]	87.67 [85–89]	91.0 [90–100]	85.67 [85–89]
Animation (AN)	83.25 [80–84]	84.25 [80–84]	86.75 [85–89]	84.25 [85–89]
Game development (GD)	84.75 [85–89]	88.25 [85–89]	93.0 [90–100]	91.50 [90–100]
Information technology (IT)	88.07 [85–89]	88.30 [85–89]	88.57 [85–89]	87.79 [85–89]

The usability of the system received favorable feedback. More than three-quarters (85.7%) of the respondents believed that it is easy to move from one module to another. 81% reported that they feel comfortable using the software. More than half of the respondents (61.9%) strongly agreed that the instructions and information presented were easy to understand. The visual appearance and responsiveness (i.e., the output is quickly displayed) of the software were also well-received, where 57.1% strongly agreed on these criteria. Most of the respondents (69.1%) strongly agreed that the software only requires minimal effort to use. Moreover, the software provided recommendations without minimal effort from the users (57.1%), and the majority (85.7%) perceived that the system performed reliably.

The recommendation of the software in terms of relevance and trust was assessed. 71.5% agreed that the output of the software was aligned with their interests and goals. More than three-quarters (76.2%) trusted the output of the software. The statements of the three students were consistent, agreeing on the innovativeness and helpfulness of the software in helping them choose computing degree programs. 76% of the users considered the recommendation reliable and helpful. When asked whether they would follow the recommendation, 62% reported that they would consider the recommendation. This indicates a relatively high consideration to act based on the software recommendation.

Two-thirds of the users (66.7%) strongly agreed that they would recommend it to their peers, and the same percentage expressed overall satisfaction. Moreover, 76% of the respondents reported that the software fulfilled its intended purpose, and more than half (52.4%) indicated they would continue using it. Furthermore, 47.6% stated that the software would help them improve their decision-making in choosing computing degree programs, while over 50% agreed that the software positively influenced their perception of computing degree program selection. These findings indicate the role of the software in guiding students toward informed computing degree program choices.

The accuracy of the system was calculated at 81.6%. Precision was 77.9%, while recall was 84.2%. These performance metrics are all higher than those reported by Baskota and Ng (2018) (accuracy = 61.6%, precision = 61.2%, recall = 62.6%) and Pupara et al. (2016) (accuracy = 69.03%, precision = 68.7%, recall = 65.5%). Therefore, both the subjective and objective evaluations of the system are positive across all criteria. Compared with prior studies (Lahoud et al., 2023; Kamal et al., 2024; Kaklauskas et al., 2013), CoDeRS extends previous educational recommender systems by incorporating OCEAN personality traits, hobbies, and career aspirations. Unlike prior works (Baskota and Ng, 2018; Pupara et al., 2016), it provides real-time and explainable results, thereby enhancing the transparency and interpretability of the recommendations. These capabilities help users better assess the accuracy and relevance of the outcomes.

## 4 Discussion

This study developed a computing degree recommender system based on academic and non-academic factors, and reported the evaluation of the software. The modular design of the software supported the logical flow and separation of functionalities. The students’ high usability and user satisfaction ratings showed that the software was effective in meeting its intended purpose. The positive feedback on visual design and responsiveness further disclosed that the user interface and user experience considerations were effective, thereby meeting the information needs of SHS regarding computing degree programs. It is worth noting that the explanation feedback and the dashboard-based output presentation enhanced the transparency and trustworthiness of the system-generated recommendations. This finding is aligned with the transparency and explain ability principle of AI (Larsson and Heintz, 2020).

Meanwhile, the recommender system component of the software was able to perform its intended purpose. The software was able to generate computing degree recommendations by analyzing multiple variables and matching them with computing degree programs. The manual computation of the persona of Student A validated the accuracy and reliability of the software. The strong trust rating and perceived relevance of the recommendations showed that the ML model produced contextually relevant outputs. Moreover, users were likely to follow the recommendation of the software. This reflects a concrete behavioral impact of the software. The overall perceived reliability is high, indicating consistent performance of the ML model. Therefore, this study supports previous works (Kamal et al., 2024; Fouad et al., 2025) on the appropriate attributes to consider when recommending computing degree programs.

The inclusion of both cognitive (e.g., academic performance) and non-cognitive factors (e.g., personality types, demographics, interests, etc.) shows the significance of these factors for a personalized and relevant recommendation output. The system has the potential to reflect the students’ authentic preference for computing degree programs and aspirations. In other words, the finding implies the need for a holistic set of student attributes to come up with a reliable and trusted recommendation in the context of degree recommendations. In a broader context, using diverse student attributes enables recommender systems to provide perspectives in assisting students in solving complex, real-world decision-making. Hence, the software is a promising tool and could be used as a springboard in supporting broader academic and career guidance initiatives.

The results of the study yielded expected and unexpected results. High usability and positive user feedback are expected since the modular design of the software is aligned with the expectations for a student-centered modular design. Another expected result is the perceived relevance and trust in the recommender system. Since the attributes are carefully selected based on the literature review and the dataset is well-represented by the current computing students, the software could correctly match the academic and non-academic attributes of the potential computing students. The manual validation of Student Persona A, showing consistency with the system recommendation, confirms that the algorithm works as intended. Consequently, there is evidence to show the positive influence on decision-making, willingness to recommend, and overall satisfaction with the software.

Meanwhile, there was also an unexpected result. Although over 60% of the respondents indicated that they would consider following the recommendation, about 40% were hesitant to do so. While the majority expressed trust in the recommendation, a significant portion were reluctant to follow it. This gap suggests the need to explore additional input attributes in future research, such as scholarship availability, kinship patronage, and reputation (e.g., performance in board examinations) (Bringula et al., 2016).

The degree recommender system has potential social benefits, especially for low-income families. College education can be expensive, particularly in private schools, and choosing the wrong degree program may result in inefficient use of financial resources. A mismatch between a degree program and a student’s interests, aspirations, or abilities can lead to dropping courses, transferring to another program, or even completing the program but pursuing a career unrelated to their field (Bringula et al., 2016; Contini et al., 2018; O'Keefe et al., 2011). This software has the potential to reduce such mismatches and could help families make more informed and effective educational investments.

From the perspective of AI-enabled educational transformation, the software demonstrates how AI can be operationalized as a student-centered decision-support tool that addresses the persistent issue of mismatch between students’ characteristics and degree program choices. The problem addressed by the software is consistent with the goals of AI-driven and digital transformation in higher education (Damanik et al., 2021; OECD, 2023; Borgonovi et al., 2025). Unlike many existing educational recommender systems that rely on predefined interest categories and structured inputs (Lahoud et al., 2023; Kamal et al., 2024; Shelake et al., 2025), the current software enables students to express their hobbies, interests, and career aspirations through open-ended responses. Natural language processing (NLP) techniques subsequently analyze these text responses. This approach provides a richer representation of student characteristics. The resulting information enables the software to generate more personalized recommendations. It also preserves transparency through explainable and interpretable results. Thus, the software aligns with the principles of beneficence, autonomy, and explicability in AI by supporting student well-being, preserving human decision-making authority, and providing understandable recommendations (Benavides et al., 2020). In other words, the software represents a practical example of how it can leverage educational data, AI, and human-centered design to strengthen personalized educational guidance while maintaining the ethical principles of responsible and trustworthy AI.

However, despite the potential benefits of the system, users (e.g., students, school administrators, and guidance counselors) should be aware that the software may introduce potential biases. Although the dataset used in the study contains more than 300 students, these students came from only one institution. Consequently, the recommendation outputs generated by the software may reflect institution-specific relevance, educational contexts, and student profiles. The applicability of the current findings to students from other academic disciplines, universities, regions, or cultural settings may be limited (Borgonovi et al., 2025). Thus, the recommendations of the system are based solely on the background and profiles of students from that institution. This may unintentionally reinforce bias by systematically directing students toward or away from specific computing degree programs. Future studies may enhance the generalizability of the outputs through the inclusion of datasets collected from multiple institutions and more diverse student populations.

Moreover, the findings of the study should be interpreted in connection with the limitations of the current validation strategy. As discussed previously, the primary purpose of the evaluation was to provide an initial baseline user-experience perspective to identify potential bugs and gather feedback on system features. While this validation approach provides valuable evidence regarding system functionality and user experience, it does not fully establish the effectiveness of the recommendations across a larger student population. For example, the study did not examine whether students would follow the suggested degree programs or choose entirely different ones. Lastly, as the system is scaled for broader deployment, longitudinal evaluations could be conducted to assess the accuracy and relevance of the recommendations over time (Floridi and Cowls, 2019; Karat, 1988).

Another limitation to consider is that the study utilized academic ability measures such as grades and national achievement test results. It should be noted, however, that these indicators may be influenced by factors such as school quality, access to educational resources, and learning opportunities. In some cases, students’ academic records may not fully reflect their true potential. Students’ interests, preferences, and aspirations—which are already included in the current system—may help balance this bias. In addition to these variables, it is recommended that future studies include teachers’ academic feedback as another indicator of student capability.

In summary, this study emphasizes that the output of the system is intended to guide users in their decision-making. Users should still apply human judgment when deciding which computing degree program a student ultimately chooses to pursue.

## 5 Conclusions, recommendations, and future works

This study developed and evaluated a computing degree program recommender system called CoDeRS. Multinomial Naïve Bayes algorithm, CBF, and cosine similarity were employed in the development of the recommender system engine. The software was developed using the prototyping software development model. Initial testing disclosed that the recommender system engine and the modules were successfully developed. The integration of both academic and non-academic dimensions contributes to the accuracy and reliability of the recommender system. Although the current study does not propose a novel machine learning algorithm for degree program recommendation, it demonstrates the effective integration of established algorithms to analyze multiple student attributes and provide computing degree program recommendations. Furthermore, despite the current software having inherent limitations, it still has clear potential to contribute to AI-driven and digital transformations in educational systems. Hence, CoDeRS is a practical, student-friendly tool that bridges technology with personalized academic advising.

Despite its potential contributions, this study has several limitations that future research could address. It is recommended that other socio-economic and external factors (e.g., scholarship availability, kinship patronage, peer suggestions, and career prospects) be incorporated into the recommender system. Future studies may also explore more advanced models, such as hybrid filtering and deep learning, to overcome the limitations of individual methods. Moreover, it is advisable to deploy the system in diverse academic contexts and institutions to ensure generalizability, cultural adaptability, and robustness across different student profiles. The software will be able to process this new data, enabling it to further fine-tune its performance. Researchers are likewise encouraged to adapt the methods used in this study when replicating it for other degree programs.

System-level performance and broader validation strategies can also be considered. Besides system functionality, other dimensions of usability (e.g., learnability, efficiency, accessibility, and dependability) and user-centered evaluation (e.g., intention to reuse, perceived utility, and trust) are worth investigating. Thinking-aloud evaluation sessions may also be conducted to capture users’ real-time experiences and perspectives. Finally, a longitudinal study could be conducted to assess the long-term impact of the software by tracking whether students pursue the recommended programs and evaluating their academic success.

## Data Availability

The datasets presented in this article are not readily available because of the Data Privacy Law in the Philippines. Requests to access the datasets should be directed to rex.bringula@liu.se.

## References

[ref1] Abay-Abay L. E. Badion W. A. Lopez J. M. Mangahas M. E. Ramiro A. Sengco B. T. (2024). Parental involvement and its effect on college students’ academic motivation and self-concept. Psychol. Educ. 18, 235–246. doi: 10.5281/zenodo.10871487

[ref2] Alsanad A. (2022). An improved Arabic sentiment analysis approach using optimized multinomial naïve Bayes classifier. Int. J. Adv. Comput. Sci. Appl. 13, 94–98. doi: 10.14569/IJACSA.2022.0130812

[ref3] Amatriain X. Pujol J. M. (2015). “Data mining methods for recommender systems,” in Recommender Systems Handbook, eds. Ricci F. Rokach L. Shapira B. (New York: Springer), 227–262.

[ref4] Banik R. (2018). Hands-On Recommendation Systems with Python: Start Building Powerful and Personalized Recommendation Engines with Python. Birmingham: Packt Publishing.

[ref5] Baskota A. Ng Y.-K. (2018). “A graduate school recommendation system using the multi-class support vector machine and KNN approaches,” in Proc. IEEE Int. Conf. Inf. Reuse Integr. (IRI), (New York: IEEE).

[ref6] Benavides L. M. C. Tamayo Arias J. A. Arango Serna M. D. Branch Bedoya J. W. Burgos D. (2020). Digital transformation in higher education institutions: a systematic literature review. Sensors 20:3291. doi: 10.3390/s20113291, 32526998 PMC7309098

[ref7] Berlingieri F. Diegmann A. Sprietsma M. (2023). Preferred field of study and academic performance. Econ. Educ. Rev. 95:102409. doi: 10.1016/j.econedurev.2023.102409

[ref8] Borgonovi F. Bastagli F. Ochojska M. Piumatti G. (2025). AI Adoption in the Education system: International Insights and Policy Considerations for Italy. OECD Artificial Intelligence Papers Available online at: https://read.oecd-ilibrary.org/en/publications/ai-adoption-in-the-education-system_69bd0a4a-en.html (Accessed June 11, 2026).

[ref9] Bringula R. P. Torres R. Merida R. A. (2016). Push and pull of institutional image indicators and computing degree programs viewed through the lens of shifters and transferees at the University of the East, Manag. Educ. Int. J. 16, 13–27. doi: 10.18848/2327-8005/CGP/v16i03/13-27

[ref10] Brown S. D. Hirschi A. (2013). “Personality, career development, and occupational attainment,” in Career Development and Counseling: Putting Theory and Research to Work, ed. Lent R. W. (Hoboken: John Wiley & Sons).

[ref11] Casanova J. R. Cervero A. Núñez J. C. Almeida L. S. Bernardo A. (2018). Factors that determine the persistence and dropout of university students. Psicothema 4, 408–414. doi: 10.7334/psicothema2018.155, 30353842

[ref12] Contini D. Cugnata F. Scagni A. (2018). Social selection in higher education: enrolment, dropout and timely degree attainment in Italy. High. Educ. 75, 785–808. doi: 10.1007/s10734-017-0170-9

[ref13] Cruz A. S. (2021). A Qualitative Study on the Factors Influencing Degree program Choices among University Students in Metro Manila. Quezon: University of the Philippines.

[ref14] Damanik F. J. Setyohadi D. B. Conf I. O. P. (2021). Analysis of public sentiment about covid-19 in indonesia on twitter using multinomial naive Bayes and support vector machine. Environ. Sci. 704:012027. doi: 10.1088/1755-1315/704/1/012027, 42216076

[ref15] DeLone W. H. McLean E. R. (2003). The DeLone and McLean model of information systems success: a ten-year update. J. Manag. Inf. Syst. 19, 9–30. doi: 10.1080/07421222.2003.11045748

[ref16] Department of Education, Policy guidelines on classroom assessment for the K to 12 basic education program, (2015). Available online at: https://www.deped.gov.ph/wp-content/uploads/2015/04/DO_s2015_08.pdf (accessed 26 June 2025).

[ref17] Floridi L. Cowls J. (2019). A unified framework of five principles for AI in society. Harvard Data Sci Rev 1:8cd550d1. doi: 10.1162/99608f92.8cd550d1, 41640455

[ref18] Fouad O. Fouad R. Hussen N. Abuhadrous I. (2025). A comprehensive review of music recommendation systems. Adv. Sci. Technol. J. 2, 1–18. doi: 10.21608/astj.2025.342474.1017

[ref19] Gill T. Vidal Rodeiro C. Zanini N. (2018). Higher education choices of secondary school graduates with a science, technology, engineering or mathematics (STEM) background. J. Furth. High. Educ. 42, 998–1014. doi: 10.1080/0309877X.2017.1332358

[ref20] John O. P. Srivastava S. (1999). “The big five trait taxonomy: history, measurement, and theoretical perspectives,” in Handbook of Personality: Theory and Research, eds. Pervin L. A. John O. P. (New York: Guilford Press).

[ref21] Julaihi N. H. Mohamadin M. I. (2024). Factors influencing Malaysian students' choice of diploma programs: analysing their interrelation with interest and satisfaction. Int. J. Serv. Manag. Sustain. 9, 23–40. doi: 10.24191/ijsms.v9i2.24205

[ref22] Kaklauskas A. Zavadskas E. K. Seniut M. Stankevič V. Raistenskis J. Simkevičius C. . (2013). Recommender system to analyze student’s academic performance. Expert Syst. Appl. 40, 6150–6165. doi: 10.1016/j.eswa.2013.05.034

[ref23] Kamal N. Sarkar F. Rahman A. Hossain S. Mamun K. A. (2024). Recommender system in academic choices of higher education: a systematic review. IEEE Access 12, 35475–35501. doi: 10.1109/ACCESS.2024.3368058

[ref24] Karat J. (1988). “Handbook of human-computer interaction,” in Handbook of Software Evaluation Methodologies, eds. Baker W. E. Landauer T. K. Marshall C. R. Pew R. W. Shackel B. (Amsterdam: Elsevier), 891–903.

[ref25] Kramer A. Bhave D. P. Johnson T. D. (2014). Personality and group performance: the importance of personality composition and work tasks. Pers. Individ. Dif. 58, 132–137. doi: 10.1016/j.paid.2013.10.019

[ref26] Lahoud C. Moussa S. Obeid C. Khoury H. E. Champin P. A. (2023). A comparative analysis of different recommender systems for university major and career domain guidance. Educ. Inf. Technol. 28, 8733–8759. doi: 10.1007/s10639-022-11541-3

[ref27] Larsson S. Heintz F. (2020). Transparency in artificial intelligence, internet. Pol. Rev. 9, 1–16. doi: 10.14763/2020.2.1469, 42321146

[ref28] Maryani H. P. Gaol F. L. Hidayanto A. N. (2022). Comparison of the system development life cycle and prototype model for software engineering. Int. J. Emerg. Technol. Adv. Eng. 12, 155–162. doi: 10.46338/ijetae0422_19, 24790640

[ref29] Mattiello H. Wittberg V. (2024). Edu-Tech Mirrors Culture as an Infrastructure Solution for Future-Oriented Engineering Edu-Tech Challenges in the Digital Transformation and Industry 5.0/Society 6.0 era via X.0 Wave/Age Theory. New York: IEEE global engineering education conference (EDUCON).

[ref30] OECD (2023). Digital Education Outlook 2023: Towards an Effective Digital Education Ecosystem. Paris: OECD Publishing.

[ref31] O'Keefe M. Laven G. Burgess T. (2011). Student non-completion of an undergraduate degree: wrong program selection or part of a career plan? High. Educ. Res. Dev. 30, 165–177. doi: 10.1080/07294360.2010.512630

[ref32] Owusu M. Owusu A. Fiorgbor E. T. Atakora J. (2021). Career aspiration of students: the influence of peers, teachers, and parents. J. Educ. Soc. Behav. Sci. 34, 67–79. doi: 10.9734/jesbs/2021/v34i230306, 42322158

[ref33] Parreño S. J. (2023). School dropouts in the Philippines: causes, changes, and statistics. Sapienza Int. J. Interdiscip. Stud. 4:e23002. doi: 10.51798/SIJIS.V4I1.552

[ref34] Person A. E. Rosenbaum J. E. Deil-Amen R. (2006). Student planning and information problems in different college structures. Teach. Coll. Rec. 108, 374–396. doi: 10.1111/j.1467-9620.2006.00655.x

[ref35] Pupara K. Nuankaew W. Nuankaew P., An institution recommender system based on student context and educational institution in a mobile environment, in: Proc. Int. Comput. Sci. Eng. Conf. (ICSEC), New York: IEEE, (2016).

[ref36] Rafanan R. J. De Guzman C. Y. (2020). Pursuing STEM careers: perspectives of senior high school students, Particip. Educ. Res. 7, 38–58. doi: 10.17275/per.20.34.7.3

[ref37] Rico-Briones E. Bueno D. C. (2019). Factors affecting the decision of first year students in choosing their degree program and school. Inst. Multidiscip. Res. Dev. J. 2, 130–135. doi: 10.13140/RG.2.2.25060.01921

[ref38] Shelake V. M. Dmello R. R. Dsouza J. J. Kadu J. J. Deshmukh S. (2025). DishaDoot: career navigation platform. Eur. J. Sustain. Dev. Res. 9:em0324. doi: 10.29333/ejosdr/16679

[ref39] Skatova A. Ferguson E. (2014). Why do different people choose different university degrees? Motivation and the choice of degree. Front. Psychol. 5:1244. doi: 10.3389/fpsyg.2014.01244, 25431561 PMC4230040

[ref40] Vedel A. (2016). Big five personality group differences across academic majors: a systematic review. Pers. Individ. Dif. 92, 1–10. doi: 10.1016/j.paid.2015.12.011

